# Individual, Environmental, and Meteorological Predictors of Daily Personal Ultraviolet Radiation Exposure Measurements in a United States Cohort Study

**DOI:** 10.1371/journal.pone.0054983

**Published:** 2013-02-06

**Authors:** Elizabeth Khaykin Cahoon, David C. Wheeler, Michael G. Kimlin, Richard K. Kwok, Bruce H. Alexander, Mark P. Little, Martha S. Linet, Daryl Michal Freedman

**Affiliations:** 1 Radiation Epidemiology Branch, Division of Cancer Epidemiology and Genetics, National Cancer Institute, National Institute of Health, Department of Health and Human Services, Bethesda, Maryland, United States of America; 2 Department of Biostatistics, School of Medicine, Virginia Commonwealth University, Richmond, Virginia, United States of America; 3 AusSun Research Lab, Institute of Health and Biomedical Innovation, Queensland University of Technology, Brisbane, Australia; 4 Epidemiology Branch, National Institute of Environmental Health Sciences, Research Triangle Park, North Carolina, United States of America; 5 Division of Environmental Health Sciences, University of Minnesota School of Public Health, Minneapolis, Minnesota, United States of America; Faculdade de Medicina, Universidade de São Paulo, Brazil

## Abstract

**Background:**

Individual exposure to ultraviolet radiation (UVR) is challenging to measure, particularly for diseases with substantial latency periods between first exposure and diagnosis of outcome, such as cancer. To guide the choice of surrogates for long-term UVR exposure in epidemiologic studies, we assessed how well stable sun-related individual characteristics and environmental/meteorological factors predicted daily personal UVR exposure measurements.

**Methods:**

We evaluated 123 United States Radiologic Technologists subjects who wore personal UVR dosimeters for 8 hours daily for up to 7 days (N = 837 days). Potential predictors of personal UVR derived from a self-administered questionnaire, and public databases that provided daily estimates of ambient UVR and weather conditions. Factors potentially related to personal UVR exposure were tested individually and in a model including all significant variables.

**Results:**

The strongest predictors of daily personal UVR exposure in the full model were ambient UVR, latitude, daily rainfall, and skin reaction to prolonged sunlight (R^2^ = 0.30). In a model containing only environmental and meteorological variables, ambient UVR, latitude, and daily rainfall were the strongest predictors of daily personal UVR exposure (R^2^ = 0.25).

**Conclusions:**

In the absence of feasible measures of individual longitudinal sun exposure history, stable personal characteristics, ambient UVR, and weather parameters may help estimate long-term personal UVR exposure.

## Introduction

Solar ultraviolet radiation (UVR) represents the strongest environmental risk factor for the development of most skin cancers [1]. A number of experimental and epidemiological observational studies have identified and assessed both the harmful and beneficial effects of UVR exposure on human health. Deleterious effects include melanoma, basal and squamous cell carcinomas, photodermatoses and actinic keratoses, eye diseases such as cataracts, and immunosuppression. However, long-term exposure to UVR is suspected to protect against certain autoimmune disorders such as multiple sclerosis, type 1 diabetes and rheumatoid arthritis [2] as well as cancers of the colon, breast, prostate, ovary, bladder, and non-Hodgkin’s lymphoma, possibly mediated through production of vitamin D [3], [4], [5], [6].

Studies of the relationship between UVR and serious health outcomes with substantial latency periods between first exposure and diagnosis of disease are hampered by the infeasibility of measuring extended individual-level exposure to solar radiation. Small observational studies have used diaries of time outdoors and personal UVR dosimeters to prospectively measure exposure [7], [8], [9], [10], [11], [12], but these methods are not feasible for measuring the extended UVR exposure relevant to diseases such as cancer. As a result, epidemiological research of UVR induced diseases typically rely on retrospective, self-reported time outdoors, static ecological-type variables such as latitude of residence, or UV indices as surrogates for long-term personal UVR exposure [5], [13], [14], [15], [16], [17], [18], [19]. These methods, however, present substantial limitations because of either only poor-to-fair reproducibility or untested validity [20], [21], [22].

This study explores an alternative approach by examining the value of individual characteristics that are stable and thus likely to be reproducible, as well as objective environmental and meteorological indices that reflect UVR as it changes over time. Several recent epidemiological studies have used satellite data, such as measures of ambient UVR, to provide estimates that also take into account time of year, elevation, and cloud cover at a particular location [23], [24], [25], [26], . In addition to satellite data, a number of databases collect localized meteorological parameters such as temperature and rainfall. In addition to influencing ambient UVR [30], weather may impact personal UVR exposure by affecting an individual’s proclivity to spend time outdoors [31]. However, ambient UVR and weather variables have not been evaluated against objectively-measured personal UVR exposure.

This study is based on a sample of participants from the United States Radiologic Technologists (USRT) cohort who completed questionnaires on demographic characteristics, location of residence, lifestyle factors, health conditions, sun sensitivity, and wore personal UVR polysulfone film dosimeters for up to 7 days for 8 hours per day [32]. A previous analysis in this group focused on agreement between self-reported time outdoors and personal UVR exposure measured from these dosimeters, but did not examine the full range of stable individual characteristics potentially relevant to personal UVR exposure (e.g., sun sensitivity) and often available in cohort studies. Nor did the analysis evaluate environmental factors linked to location of residence [32].

The objective of the current analysis was to evaluate the impact of stable individual characteristics and environmental/meteorological factors on personal UVR exposure so as to guide the choice of surrogates for long-term UVR exposure in epidemiologic studies. The present study is the first to assess the contributions of weather parameters and Geographical Information System (GIS) satellite estimates of ambient UVR in addition to individual characteristics related to sun sensitivity as determinants of daily personal UVR exposure.

## Materials and Methods

### Study Population

This study uses data from a sample of subjects in the United States Radiologic Technologists (USRT) study, a cohort comprised of radiologic technologists living in the United States who were certified by the American Registry of Radiologic Technologists for at least 2 years between 1926 and 1982. Details of the USRT cohort have been previously described [33], [34]. To be included in this study, subjects had to have completed a self-administered questionnaire from 2003 to 2005 and worn a personal UVR dosimeter for up to 7 days in 2004. Volunteers were selected so that approximately equal numbers were split between residents of northern U.S. latitudes (Minnesota and Wisconsin) and southern latitudes (North Carolina and Georgia), men and women, and two age groups (40 to 59 years; 60 years or older) [32]. Among the 300 individuals randomly selected for recruitment, 127 agreed to participate, and 123 subjects satisfied the inclusion criteria for the study sample. Due to 24 missing daily personal UVR exposure values, the final sample contained a total of 837 daily personal UVR exposure measurements.

### Ethics Statement

The USRT Study has been approved annually by the human subjects review boards at the University of Minnesota and the National Cancer Institute and subjects gave their written, informed consent.

### Personal UVR Exposure Measurements

Personal solar UVR exposure was measured using polysulfone film dosimeters, which, through a change in optical characteristics, represent the UVR exposure received. Participants wore a dosimeter on their left shoulder attached to the outside of their clothing from 9:00 A.M. to 5:00 P.M. each day during a consecutive 7 day period between September 1^st^ and October 5^th^ of 2004. Dosimeters were developed specifically for the study and have been previously described [32]. They were calibrated to the solar spectrum for each location using surface UV irradiances from UVB monitoring stations of the U.S. Department of Agriculture, with an error estimated to be on the order of 10% [35]. To examine reproducibility, 14 individuals wore a second dosimeter placed next to the first dosimeter during the 7 day period. When the readings of the two dosimeter measurements were compared, a high level of correlation (Pearson r = 0.92, P<0.001) was obtained.

### Individual Factors

Information about potential individual predictors of personal UVR came from the third survey of USRT participants who completed self-administered questionnaires between 2003 and 2005. These questionnaires ascertained basic demographic information (age, sex, education, marital status, and race), location of residence, weight, height, smoking history, history of specific cancers, other serious health conditions, and sun exposure-related characteristics (hair and eye color, complexion, skin reaction to sunlight, and sunburn history).

### Environmental and Meteorological Factors

Information about potential environmental predictors of personal UVR came from two nationwide databases: 1) daily ambient UVR using the Total Ozone Mapping Spectrometer database maintained by the National Aeronautics and Space Administration (NASA) [36] and 2) meteorological data collected by numerous airports across the country and maintained by the National Oceanic and Atmospheric Administration [37]. UVR exposures were determined by linking the residential addresses during the study period reported by respondents with cloud-adjusted daily ambient erythemal UVR, which is weighted more heavily towards the UVB side of the UV spectrum. This is provided by NASA on a 1 degree latitude by 1.25 degree longitude grid. Weather variables including temperature, rainfall, dew point, relative humidity, and wind speed were collected hourly for the days corresponding to the study period for each participant. The hourly values were averaged for the hours between 9:00 A.M. and 5:00 P.M. for temperature, dew point, relative humidity, and wind speed. Rainfall was summed for these hours. For each participant, daily exposure to weather parameters and to daily ambient UVR was assigned using data from the nearest airport and TOMS grid cell, respectively, using ArcGIS 9.1 software (ESRI 2005).

### Statistical Analysis

All available dosimeter exposure measurements (for up to 7 days) were averaged for each person to create an average daily personal UVR measure. Means and medians of average daily personal UVR exposure were calculated per person across the individual-level factors. Since average daily personal UVR was not normally distributed, differences in the distribution of personal UVR exposure across these factors were tested using the Kruskal-Wallis test of heterogeneity of medians and trends were tested through linear regression models treating ordinal variables as continuous and using continuous age and BMI.

In all regression models, we used the natural log transformation of personal UVR which resulted in normally distributed residuals. We used random intercept models to account for correlation of errors stemming from repeated measures over the week from the same subject. A log-linear random intercept regression model for personal UVR can be expressed as:
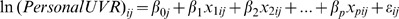
where i represents day, j represents subject, p is the number of predictors, β_0j_ is the subject-specific random intercept, x_1ij_, x_2ij_, etc. are independent predictors, and ε_ij_ represents the random measurement error which is ∼Normal(0,σ^2^). For ease of interpretation, coefficients from the regression which represent change in ln(Personal UVR) between one category and the reference category for a predictor were converted to % changes (PC) according to the equation,



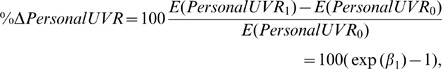
where *E(PersonalUVR_1_)* is the expected personal UVR exposure for an individual subject in category 1 of some factor and *E(PersonalUVR_0_)* is the expected personal UVR exposure for the reference category [38]. This unitless measure represents the % change in the geometric mean of personal UVR comparing one category of a factor to the reference category of that factor after adjusting for all other factors.

Individual and environmental factors related to daily personal UVR exposure were tested individually and in a model including all significant factors. Interaction terms and linear splines of continuous variables were also considered for inclusion into multiple regression models. Forward selection was used to select the variables most strongly associated with personal UVR exposure (by adding variables one at a time and retaining those that were statistically significant based on p-values from type III F tests). Backward selection yielded the same models for both full and environmental only models.

A cross-validation was performed to illustrate the performance of the proposed model in independent samples. Based on forward selection in the full dataset, we chose 4 nested models containing significant predictors. We then created 10 random sets of 2/3-1/3 split of the participants after stratifying on sex and north/south. The 2/3 samples were used to fit the 4 models. Based on the coefficients from these models, we predicted log doses for the remaining 1/3 of participants. The mean squared error for these models were calculated based on the difference between predicted and observed, and then averaged over all ten 1/3 sets.

To estimate the proportion of variability accounted for by key variables, we computed R^2^ values by calculating the percent change in total variance of specified models from a null model using the method of Snijders and Bosker for random intercept models [39]. Tests were two-sided and P values were considered significant at the 0.05 alpha level Kruskal-Wallis tests were performed using the NPAR1WAY procedure and regression analyses were conducted with the MIXED procedure using SAS software V9.2 (SAS Institute, Inc.).

## Results

The distribution of average daily personal UVR exposure (averaged across all available daily measurements per participant over a week) varied significantly across several individual characteristics (Table 1). Median average daily personal UVR exposure was significantly higher in men than in women (P = 0.01) and decreased for increasing BMI (though no statistically significant trend was observed for BMI). Median average daily personal UVR varied across levels of several constitutional characteristics that relate to sun sensitivity. Participants with red or blonde hair, light complexion, severe or painful sunburn from 30 minutes of sunlight, and not tanning when exposed to prolonged sunlight recorded lower median average daily personal UVR exposure than their less UVR-sensitive counterparts. Factors that were not significantly related to the median of average daily personal UVR included age, education, marital status, smoking, eye color, having a potentially disabling condition, history of skin cancer, or history of blistering sunburns.

**Table 1:** Distribution of average daily personal UVR (J/m^2^) across individual factors for 123 participants from the United Radiologic Technologists’ study.^a^

		N	Mean UVR	Median UVR	P-value^b^	P for trend^c^
**Age**						
	40 to 49	13	126.55	86.09	0.85	0.33
	50 to 59	46	131.73	78.77		
	60 and greater	64	139.98	88.55		
**Sex**						
	Male	58	171.24	94.88	0.01	
	Female	65	103.56	70.85		
**Highest education completed**						
	X-ray tech program	65	109.7	72.52	0.06	
	College/graduate school	58	164.35	96.87		
**Marital status**						
	Married	103	136.78	86.09	0.61	
	Unmarried	19	126.67	72.52		
	Missing	1	168.52	168.52		
**Race**						
	White (non-Hispanic)	122	129.63	83.22	0.09	
	Black	1	848.33	848.33		
**BMI**						
	Normal, 18.5–24.9	36	158.71	98.01	0.04	0.07
	Overweight, 25–29.9	50	142.76	92.8		
	Obese, ≥30	34	102.63	42.76		
	Missing	3	107.35	86.09		
**Current smoker**						
	Yes	10	185.47	75.96	0.70	
	No	112	131.82	85.08		
	Missing	1	45.01	45.01		
**Previous skin cancer**						
	Yes	13	145.46	99.8	0.39	
	No	110	134.29	81.69		
**Potentially disabling condition** d						
	Yes	32	127.72	97.54	0.68	
	No	91	138.2	77.14		
**Eye color**						
	Blue/green/grey	76	130.25	81.69	0.26	0.33
	Hazel	22	120.39	63.72		
	Brown	24	167.27	140.15		
	Missing	1	101.52	101.52		
**Hair color at age 20**						
	Red or blonde	17	68.97	67.21	0.02	<0.01
	Light/medium brown	68	127.07	85.08		
	Dark brown or black	37	182.39	127.58		
	Missing	1	101.52	101.52		
**Complexion**						
	Light	46	95.59	68.75	0.02	<0.01
	Medium	67	153.89	95.1		
	Dark	7	255.08	232.98		
	Missing/Other	3	56.7	66.02		
**Skin reaction to 30 minutes of sunlight**						
	Severe/painful sunburn	31	92.03	84.06	0.01	<0.01
	Mild sunburn	76	131.4	75.47		
	Tanned, no sunburn	14	205.3	201.87		
	Unknown	1	101.52	101.52		
	No change in skin color	1	848.33	848.33		
**Skin reaction to prolonged sunlight**						
	Deeply tanned	34	179.38	95.2	0.06	<0.01
	Moderately tanned	58	120.99	80.7		
	Lightly tanned	26	95.19	74.31		
	Not tanned	3	40.75	19.78		
	Missing	2	474.93	474.93		
**Ever had blisters from sunburn**						
	Yes	73	133.61	90.31	0.76	
	No	49	138.94	80.4		
	Missing	1	101.52	101.52		

a Daily UVR values averaged over the week for each of 123 participants.

b P-values from Kruskal-Wallis test of heterogeneity of medians.

c P for trend from log-linear regression with continuous age and BMI.

d Includes arthritis, osteoporosis, multiple sclerosis, scleroderma, and lupus.

**Figure 1:**
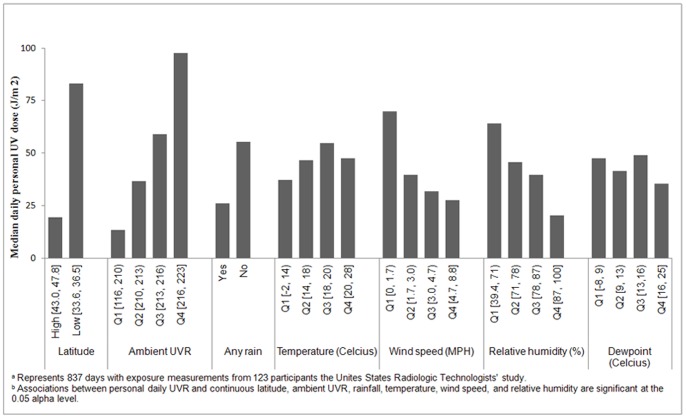
Distribution of personal UVR across environmental and meteorological factors. ^a^Represents 837 days with exposure measurements from 123 participants the Unites States Radiologic Technologists’ study. ^b^Associations between personal daily UVR and continuous latitude, ambient UVR, rainfall, temperature, wind speed, and relative humidity are significant at the 0.05 alpha level.

The distribution of daily personal UVR exposure also varied across several environmental and meteorological factors (Figure 1). Median daily personal UVR levels tended to be higher for latitudes closer to the equator, days with higher ambient UVR, no rain, low wind speed, and low relative humidity. Days with temperatures between 18 and 20°C (64–68°F) recorded the highest personal UVR.

Table 2 demonstrates the regression relationships of individual and environmental/meteorological factors with daily personal UVR exposures. Percent change in daily personal UVR exposure was independently associated with sex, hair color, complexion, skin reaction to 30 minutes of sunlight, skin reaction to prolonged sunlight, latitude, ambient UVR, rainfall, temperature, wind speed, and relative humidity. Latitude of residence individually explained the greatest proportion of variability in daily personal UVR exposure (R^2^ = 0.15) followed by ambient UVR (R^2^ = 0.14).

**Table 2:** Percent change in personal UVR exposure and 95% confidence intervals.

		Unadjusted models^a^				Full model (R^2^ = 0.30)^b^			Environmental model (R^2^ = 0.25)^c^		
		% Change	95% CI		R^2^	% Change	95% CI		% Change	95% CI	
**Individual factors**											
**Sex**											
Female		Ref			0.02						
Male		56.10	4.49	133.2							
**Hair color at age 20**											
Red or blonde		Ref			0.03						
Light/medium brown		41.08	−22.66	157.36							
Dark brown or black		130.34	20.63	339.81							
**Complexion**											
Light		Ref			0.05						
Medium		104.80	36.66	206.79							
Dark		197.35	25.35	605.36							
**Skin reaction to 30 minutes**											
Severe or painful sunburn		Ref			0.05						
Mild sunburn		64.43	3.88	160.26							
Tanned, no sunburn		222.22	60.84	543.53							
No change in skin color		1555.09	79.04	15200.07							
**Skin reaction to prolonged sunlight**											
Not tanned/Lightly tanned		Ref			0.04	Ref					
Moderately tanned		64.80	1.11	168.61		57.55	3.98	138.71			
Deeply tanned		158.07	49.46	345.59		164.31	65.16	322.99			
**Environmental/meteorological factors**											
**Latitude (°)**		−12.41	−15.32	−9.40	0.15	−8.72	−11.84	−5.49	−8.41	−11.79	−4.91
**Ambient UVR**		1.35	1.14	1.57	0.14	1.19	0.94	1.44	1.20	0.95	1.45
**Daily rainfall (in)**		−6.29	−8.18	−4.35	0.04	−3.09	−5.03	−1.11	−3.10	−5.04	−1.12
**Average daily temperature (**°**C)**		0.81	−1.99	3.70	0.03						
**Average daily wind speed (mph)**		−9.57	−14.89	−3.92	0.02						
**Average daily relative humidity (%)**		−2.91	−3.90	−1.92	0.02						

Abbreviations: UVR = ultraviolet radiation; CI = confidence interval; in = inches; °C = Celsius; mph = miles per hour.

a Includes factors from Table 1 and Figure 1 that are significant in regression.

b Includes factors from Table 1 and Figure 1 that are significant in regression using forward selection.

c Includes factors from Figure 1 that are significant in regression using forward selection.

In the full model, which considered individual factors from Table 1 and environmental factors from Figure 1, % change in daily personal UVR was significantly associated with ambient UVR, latitude, rainfall, and skin reaction to prolonged sunlight (R^2^ = 0.30) (Table 2). For every increase in degree of latitude, participants’ personal UVR exposure changed by −8.72% (95% CI: −11.84 to −5.49%) after adjusting for other factors. Adjusted % change of personal UVR increased with increasing unit of ambient UVR (PC = 1.19%, 95% CI: 0.94 to 1.44%) and decreased with increasing daily rainfall (PC = −3.09%, 95% CI: −5.03 to −1.11%). We found a 164.31% (95% CI: 65.16 to 322.99%) increase in personal UVR exposure for participants who deeply tanned when exposed to prolonged sunlight as compared to those who did not tan or tanned lightly.

In the environmental model, which considered factors from Figure 1, % change in daily personal UVR was significantly associated with ambient UVR, latitude, and daily rainfall after adjustment (R^2^ = 0.25) (Table 2). Adjusted % change of personal UVR increased with increasing unit of ambient UVR (PC = 1.20%, 95% CI: 0.95 to 1.45%) and decreased with increasing degree of latitude (PC = −8.41%, 95% CI: −11.79 to 4.91%) and increasing inches of rainfall (PC = −3.10%, 95% CI: −5.04 to −1.12%).

**Figure 2:**
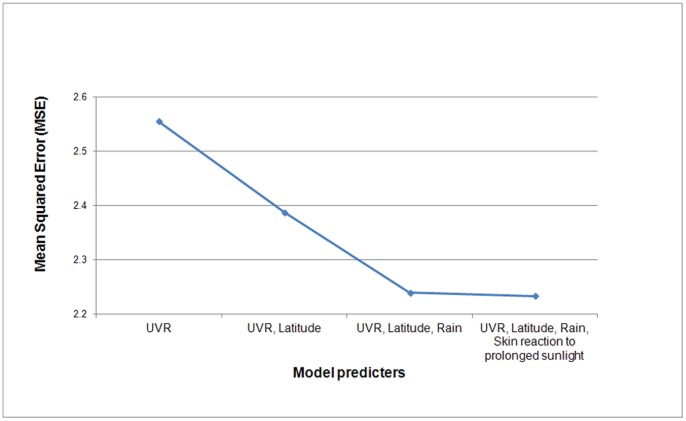
Cross validation of predicted vs. personal UVR dose for 4 nested models.

The cross-validation mean squared errors (MSE) for nested models are displayed in Figure 2. Following the inclusion of UVR, latitude, and rain into the models, the cross-validation MSE progressively reduces as successively more complicated models are fitted, suggesting that the final model, incorporating UVR, latitude, rainfall, and skin reaction, is optimal.

## Discussion

In this study, we developed multiple regression models that considered both self-reported stable individual characteristics and objective historically available environmental and meteorological factors to predict personal UVR exposure. We found that the strongest predictors of personal UVR exposure were ambient UVR, latitude, rainfall, and skin reaction to prolonged sunlight (R^2^ = 0.30). The environmental model, which depended only on residential location (for linkage to other UVR exposure-related data), included ambient UVR, latitude, and daily rainfall (R^2^ = 0.25), as the strongest predictors of daily personal UVR exposure.

Although average daily personal UVR exposure varied significantly across a number of individual characteristics, none of these characteristics were exceptional predictors of average daily personal UVR exposure. Even significant individual characteristics presented in Table 2 did not have R^2^ values greater than 0.05 (e.g., complexion), indicating that most of these individual-level characteristics explained little variance in average weekly personal UVR exposure. These results are consistent with a previous study that examined several sun sensitivity characteristics in relation to solar keratoses and severe solar elastosis, two histological measures of actinic damage [40]. The investigators found no significant trends in increased risk of either of these conditions with skin color, hair color, eye color, or skin reaction to prolonged sunlight.

Several studies have also explored the value of time-varying behavioral characteristics, such as time spent outdoors, in explaining personal UVR exposure or predicting the occurrence of sun-related disease. These have measured short-term exposure from daily diaries [32], regular time spent outdoors throughout the year using surveys [41], or cumulative exposure from sun exposure history questionnaires [20], [40]. A previous study in this group found that the correlation between time outdoors from daily diaries and personal UV doses for the same days to be 0.63 (p<0.001) and 0.72 (p<0.001) in the south and north, respectively [32]. Although these results are encouraging, daily recording of time outdoors is not feasible for long time periods. Daily records are also inapplicable to estimating retrospective UV exposures. Retrospective sun exposure history has shown poor to moderate reproducibility, so that one-time self-reports of number of lifetime sunburns or time outdoors present serious limitations for quantifying long-term UVR exposure [20], [21], [22]. A study by Rosso and colleagues revealed that reliability of self-reported sun exposure history can be associated with possible confounding factors, such as education and location of outdoor vacations during childhood [21]. An additional limitation is that reported sun exposure has shown only moderate agreement with biological markers of sun damage [20]. Given the infeasibility of collecting ongoing long-term measures of time outdoors or personal UVR exposure and the unreliability of self-reported sun exposure history, historically available environmental data may offer a useful surrogate for UVR exposure.

The current study found that environmental and meteorological variables were stronger predictors of personal UVR exposure than a number of stable individual host variables. Our R^2^ values for ambient UVR (R^2^ = 0.14) and residential latitude (R^2^ = 0.15) were similar to a study which found R^2^ = 0.16 for both ambient UVR and latitude using simulated data of facial UVR exposure [42]. We also found that both ambient UVR and latitude were significant predictors of objectively measured UVR when included in a multivariable model also containing rainfall, and contributed to a greater overall R^2^ (0.25). Since our personal UVR exposure measurements came from two main geographic regions, in addition to environmental conditions, latitude may reflect regional behaviors for time spent outdoors.

Despite the strength of the association between environmental/meteorological factors and objectively measured personal UVR, our variables only accounted for 25–30% of the variation in personal UVR exposure. Though much of the heterogeneity that remained in personal UVR exposure is likely to be explained by varying time outdoors [32], some may also be due to the fact that all individuals residing in a particular 1 degree latitude by 1.25 degree longitude grid were assigned the same ambient UVR value representing the average of that area. Misclassification of ambient UVR exposure associated with using this variable caused predominantly Berkson error, which occurs when a group average is used instead of an individual value [43]. A similar situation arose for the assignment of weather parameters, which were centered on a particular airport. A regression of these environmental variables on a given long-term health outcome should provide unbiased coefficients, though there would be an associated loss in power [43], [44]. Misclassification from using these surrogates should be taken into account when estimating the effect of sun exposure on risk of long-term health outcomes, since it can bias the relative risk toward the null.

Ecological fallacy is another potential limitation to using meteorological factors to predict individual risk. For example, some other factor associated with location may be strongly related to individual risk. However, this problem is reduced when information is collected for location of residence throughout the lifetime, assuming some subjects do not live in the same place throughout their life. The potential for ecological fallacy will depend on this and other considerations specific to the study population, exposure period, and health outcome under investigation.

Our study was strengthened by the range of latitudes of residence and to a lesser extent by the time of year participants wore personal dosimeters (September 1^st^ - October 5^th^), which provided enough variation in ambient UVR, latitude, rainfall, temperature, wind speed, and relative humidity to detect independent associations with personal UVR exposure. However, our measure of daily personal UVR is not equivalent to dose in that it does not take into account protective behaviors such as sunscreen use or clothing. We were also not able to assess how wearing a dosimeter may have influenced behavior in terms of the quantity of time spent outdoors or the particular times of day participants were outdoors. In addition, since our sample included participants from an occupational cohort of indoor healthcare workers in the United States from two specific regions of the country during a six-week period, the generalizability of our findings may not extend to other U.S. workers, general population groups, residents of other countries, or different seasons.

To guide the choice of UVR exposure surrogates in epidemiologic studies, in this report we evaluated some alternate metrics of personal UVR exposure based on relatively stable constitutional characteristics and objective environmental and meteorological factors. This type of information is often available in epidemiologic studies. Ongoing cohort studies frequently collect information on location of residence, which may be used to provide environmental information (e.g., latitude, ambient UVR, and weather data) as surrogates of personal UVR exposure prior to outcome ascertainment. Some studies may also include variables on skin sensitivity. In the absence of high quality longitudinal individual-level sun exposure history, self-reported skin reaction to prolonged sunlight, ambient UVR, and meteorological parameters may be helpful surrogates to guide future research evaluating the relationship between long-term solar radiation and health outcomes. Our findings lend additional support to the use of these long-term exposure surrogates in previous studies. Future methodological studies may examine how well individual and environmental factors predict UVR exposures among subjects with a wider range of geographic UVR exposures over multiple seasons.
